# Bis[4-(dimethyl­amino)pyridinium] tetra­bromidodiphenyl­stannate(IV)

**DOI:** 10.1107/S1600536808010830

**Published:** 2008-04-23

**Authors:** Quai Ling Yap, Kong Mun Lo, Seik Weng Ng

**Affiliations:** a Department of Chemistry, University of Malaya, 50603 Kuala Lumpur, Malaysia

## Abstract

The Sn^IV^ atom of the stannate anion in the title salt, (C_7_H_11_N_2_)_2_[SnBr_4_(C_6_H_5_)_2_], lies on a center of inversion in a tetra­gonally compressed octa­hedron. The two independent Br atoms in the anion are hydrogen-bond acceptors for the same cation.

## Related literature

For the structure of dipyridinium tetra­bromidostannate(II), see: Tuleda & Khan (1991 ▶).
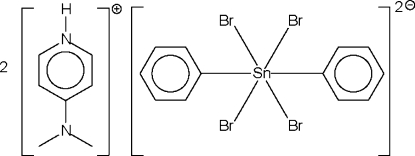


## Experimental

### 

#### Crystal data

(C_7_H_11_N_2_)_2_[SnBr_4_(C_6_H_5_)_2_]
*M* *_r_* = 838.89
Monoclinic, 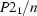

*a* = 10.7803 (2) Å
*b* = 9.3847 (2) Å
*c* = 14.4068 (4) Å
β = 94.126 (2)°
*V* = 1453.76 (6) Å^3^
*Z* = 2
Mo *K*α radiation
μ = 6.40 mm^−1^
*T* = 100 (2) K
0.24 × 0.18 × 0.12 mm

#### Data collection

Bruker SMART APEX diffractometer
Absorption correction: multi-scan (*SADABS*; Sheldrick, 1996 ▶) *T*_min_ = 0.386, *T*_max_ = 0.514 (expected range = 0.348–0.464)
11853 measured reflections
3334 independent reflections
2688 reflections with *I* > 2σ(*I*)
*R*_int_ = 0.035

#### Refinement

*R*[*F* ^2^ > 2σ(*F* ^2^)] = 0.024
*wR*(*F* ^2^) = 0.051
*S* = 0.99
3334 reflections
166 parameters
1 restraint
H atoms treated by a mixture of independent and constrained refinement
Δρ_max_ = 0.47 e Å^−3^
Δρ_min_ = −0.42 e Å^−3^

### 

Data collection: *APEX2* (Bruker, 2007 ▶); cell refinement: *SAINT* (Bruker, 2007 ▶); data reduction: *SAINT*; program(s) used to solve structure: *SHELXS97* (Sheldrick, 2008 ▶); program(s) used to refine structure: *SHELXL97* (Sheldrick, 2008 ▶); molecular graphics: *X-SEED* (Barbour, 2001 ▶); software used to prepare material for publication: *publCIF* (Westrip, 2008 ▶).

## Supplementary Material

Crystal structure: contains datablocks global, I. DOI: 10.1107/S1600536808010830/tk2264sup1.cif

Structure factors: contains datablocks I. DOI: 10.1107/S1600536808010830/tk2264Isup2.hkl

Additional supplementary materials:  crystallographic information; 3D view; checkCIF report

## Figures and Tables

**Table:** 

Sn1—C1	2.143 (3)
Sn1—Br1	2.7395 (2)
Sn1—Br2	2.7470 (3)

**Table:** 

C1—Sn1—Br1	90.53 (7)
C1—Sn1—Br1^i^	89.47 (7)
C1—Sn1—Br2	89.64 (7)
C1—Sn1—Br2^i^	90.36 (7)
Br1—Sn1—Br2	88.981 (8)
Br1—Sn1—Br2^i^	91.019 (8)

Symmetry code: (i) 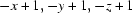.

**Table 2:** Hydrogen-bond geometry (Å, °)

*D*—H⋯*A*	*D*—H	H⋯*A*	*D*⋯*A*	*D*—H⋯*A*
N2—H2N⋯Br1	0.88 (1)	2.79 (3)	3.385 (3)	126 (3)
N2—H2N⋯Br2	0.88 (1)	2.81 (3)	3.485 (3)	135 (3)

**Fig. 1.**
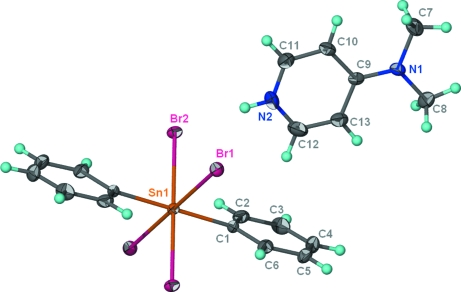
Thermal ellipsoid plot (Barbour, 2001) plot of [C7H11N]2 [SnBr4(C6H5)2] at the 70% probability level. Hydrogen atoms are drawn as spheres of arbitrary radius.

## supplementary crystallographic information

### Comment

Bis[4-(dimethylamino)pyridinium] tetrabromidodiphenylstannate(IV), (I) (Fig. 1 and Table 1) was the product of the cleavage of the mixed alkyl/triarylstannate, cyclopentyltriphenyltin, by 4-dimethylaminopyridine hydrobromide perbromide. The stannate has the tin atom in a tetragonally compressed octahedral Br_4_C_2_ environment. The anion has also been reported as the centrosymmetric pyridinium salt: Sn–Br = 2.7592 (3), 2.7737 (3) and Sn–C 2.158 (3) Å (Tuleda & Khan, 1991). Connections between ions are of the type N-H···Br (Table 2) so that each independent pair of bromide atoms are linked to the same cation.

### Experimental

Cyclopentyltriphenyltin (1.36 g, 3 mmol) and 4-dimethylaminopyridine hydrobromide perbromide (1.1 g, 3 mmol) were heated in chloroform (100 ml) for 3 h. The filtered solution when allowed to evaporate yielded large yellow crystals, m.p. 470–473 K.

### Refinement

Carbon-bound H-atoms were placed in calculated positions (C—H 0.95 to 0.98 Å) and were included in the refinement in the riding model approximation, with *U*(H) set to 1.2 to 1.5*U*_eq_(C). The ammonium H atom was refined with a distance restraint of N–H 0.88±0.01 Å; its displacement parameter was freely refined.

### Crystal data

**Table:**

(C_7_H_11_N_2_)_2_[SnBr_4_(C_6_H_5_)_2_]	*F*_000_ = 812
*M**_r_* = 838.89	*D*_x_ = 1.916 Mg m^−^^3^
Monoclinic, *P*2_1_/*n*	Mo *K*α radiation λ = 0.71073 Å
Hall symbol: -P 2yn	Cell parameters from 3449 reflections
*a* = 10.7803 (2) Å	θ = 2.3–28.3º
*b* = 9.3847 (2) Å	µ = 6.40 mm^−^^1^
*c* = 14.4068 (4) Å	*T* = 100 (2) K
β = 94.126 (2)º	Block, colorless
*V* = 1453.76 (6) Å^3^	0.24 × 0.18 × 0.12 mm
*Z* = 2	

### Data collection

**Table:**

Bruker SMART APEX diffractometer	3334 independent reflections
Radiation source: fine-focus sealed tube	2688 reflections with *I* > 2σ(*I*)
Monochromator: graphite	*R*_int_ = 0.035
*T* = 100(2) K	θ_max_ = 27.5º
ω scans	θ_min_ = 2.3º
Absorption correction: Multi-scan(SADABS; Sheldrick, 1996)	*h* = −14→14
*T*_min_ = 0.386, *T*_max_ = 0.514	*k* = −12→12
11853 measured reflections	*l* = −18→14

### Refinement

**Table:**

Refinement on *F*^2^	Secondary atom site location: difference Fourier map
Least-squares matrix: full	Hydrogen site location: inferred from neighbouring sites
*R*[*F*^2^ > 2σ(*F*^2^)] = 0.024	H atoms treated by a mixture of independent and constrained refinement
*wR*(*F*^2^) = 0.051	*w* = 1/[σ^2^(*F*_o_^2^) + (0.0228*P*)^2^] where *P* = (*F*_o_^2^ + 2*F*_c_^2^)/3
*S* = 0.99	(Δ/σ)_max_ = 0.001
3334 reflections	Δρ_max_ = 0.47 e Å^−^^3^
166 parameters	Δρ_min_ = −0.42 e Å^−^^3^
1 restraint	Extinction correction: none
Primary atom site location: structure-invariant direct methods	

### Fractional atomic coordinates and isotropic or equivalent isotropic displacement parameters (Å^2^)

**Table:**

	*x*	*y*	*z*	*U*_iso_*/*U*_eq_	
Sn1	0.5000	0.5000	0.5000	0.01159 (7)	
Br1	0.25635 (2)	0.50711 (3)	0.43147 (2)	0.01591 (7)	
Br2	0.55014 (2)	0.71661 (3)	0.38004 (2)	0.01549 (7)	
N1	0.05057 (19)	1.2090 (2)	0.45413 (16)	0.0168 (5)	
N2	0.2527 (2)	0.8608 (3)	0.3851 (2)	0.0309 (7)	
H2N	0.296 (3)	0.785 (2)	0.372 (3)	0.057 (12)*	
C1	0.4622 (2)	0.6546 (3)	0.60374 (19)	0.0130 (6)	
C2	0.5344 (2)	0.7784 (3)	0.6151 (2)	0.0185 (6)	
H2	0.6043	0.7916	0.5794	0.022*	
C3	0.5038 (3)	0.8816 (3)	0.6784 (2)	0.0232 (7)	
H3	0.5529	0.9654	0.6862	0.028*	
C4	0.4025 (3)	0.8631 (3)	0.7301 (2)	0.0248 (7)	
H4	0.3807	0.9354	0.7722	0.030*	
C5	0.3325 (3)	0.7406 (3)	0.7212 (2)	0.0219 (7)	
H5	0.2639	0.7277	0.7582	0.026*	
C6	0.3615 (2)	0.6360 (3)	0.65865 (19)	0.0172 (6)	
H6	0.3131	0.5515	0.6530	0.021*	
C7	−0.0284 (3)	1.2845 (3)	0.3844 (2)	0.0260 (7)	
H7A	0.0236	1.3368	0.3429	0.039*	
H7B	−0.0816	1.3517	0.4153	0.039*	
H7C	−0.0804	1.2160	0.3481	0.039*	
C8	0.0588 (3)	1.2631 (3)	0.5493 (2)	0.0209 (6)	
H8A	0.0386	1.1866	0.5920	0.031*	
H8B	−0.0001	1.3418	0.5541	0.031*	
H8C	0.1435	1.2972	0.5657	0.031*	
C9	0.1188 (2)	1.0979 (3)	0.4305 (2)	0.0141 (6)	
C10	0.1155 (2)	1.0461 (3)	0.3381 (2)	0.0182 (6)	
H10	0.0668	1.0937	0.2900	0.022*	
C11	0.1821 (3)	0.9282 (3)	0.3182 (2)	0.0254 (7)	
H11	0.1786	0.8932	0.2562	0.031*	
C12	0.2617 (3)	0.9093 (3)	0.4731 (2)	0.0280 (8)	
H12	0.3141	0.8609	0.5186	0.034*	
C13	0.1979 (2)	1.0250 (3)	0.4982 (2)	0.0201 (7)	
H13	0.2058	1.0577	0.5607	0.024*	

### Atomic displacement parameters (Å^2^)

**Table:**

	*U*^11^	*U*^22^	*U*^33^	*U*^12^	*U*^13^	*U*^23^
Sn1	0.01150 (12)	0.01286 (14)	0.01055 (14)	0.00047 (10)	0.00166 (10)	0.00068 (11)
Br1	0.01193 (13)	0.01943 (15)	0.01628 (15)	0.00059 (10)	0.00046 (10)	0.00031 (12)
Br2	0.01807 (13)	0.01534 (14)	0.01326 (14)	−0.00163 (11)	0.00254 (10)	0.00332 (13)
N1	0.0165 (11)	0.0180 (12)	0.0155 (13)	0.0016 (10)	−0.0018 (9)	0.0031 (11)
N2	0.0372 (15)	0.0258 (16)	0.0305 (17)	0.0166 (13)	0.0093 (13)	0.0049 (14)
C1	0.0147 (13)	0.0135 (14)	0.0109 (15)	0.0026 (11)	0.0011 (11)	−0.0007 (12)
C2	0.0209 (14)	0.0194 (15)	0.0155 (15)	−0.0009 (12)	0.0027 (12)	0.0045 (13)
C3	0.0308 (16)	0.0152 (16)	0.0227 (17)	−0.0012 (12)	−0.0039 (13)	−0.0025 (13)
C4	0.0339 (17)	0.0243 (17)	0.0158 (16)	0.0109 (14)	−0.0004 (13)	−0.0078 (14)
C5	0.0193 (14)	0.0345 (19)	0.0120 (15)	0.0065 (12)	0.0012 (12)	−0.0018 (14)
C6	0.0139 (13)	0.0235 (16)	0.0138 (15)	0.0001 (11)	−0.0015 (11)	−0.0003 (13)
C7	0.0217 (15)	0.0307 (18)	0.0244 (17)	0.0081 (13)	−0.0072 (13)	−0.0020 (15)
C8	0.0233 (14)	0.0217 (16)	0.0178 (16)	−0.0016 (12)	0.0018 (12)	−0.0022 (13)
C9	0.0132 (12)	0.0147 (14)	0.0147 (15)	−0.0048 (10)	0.0016 (11)	0.0038 (12)
C10	0.0184 (14)	0.0163 (15)	0.0197 (16)	0.0006 (11)	0.0000 (12)	0.0056 (13)
C11	0.0345 (17)	0.0237 (17)	0.0188 (17)	0.0052 (14)	0.0073 (14)	0.0021 (15)
C12	0.0271 (16)	0.0303 (19)	0.0261 (19)	0.0057 (14)	−0.0014 (14)	0.0102 (16)
C13	0.0192 (13)	0.0234 (17)	0.0175 (16)	−0.0012 (12)	0.0010 (12)	0.0045 (13)

### Geometric parameters (Å, °)

**Table:**

Sn1—C1	2.143 (3)	C4—H4	0.9500
Sn1—C1^i^	2.143 (3)	C5—C6	1.384 (4)
Sn1—Br1	2.7395 (2)	C5—H5	0.9500
Sn1—Br1^i^	2.7395 (2)	C6—H6	0.9500
Sn1—Br2	2.7470 (3)	C7—H7A	0.9800
Sn1—Br2^i^	2.7470 (3)	C7—H7B	0.9800
N1—C9	1.334 (3)	C7—H7C	0.9800
N1—C7	1.454 (3)	C8—H8A	0.9800
N1—C8	1.459 (4)	C8—H8B	0.9800
N2—C12	1.344 (4)	C8—H8C	0.9800
N2—C11	1.342 (4)	C9—C10	1.415 (4)
N2—H2N	0.879 (10)	C9—C13	1.424 (4)
C1—C6	1.399 (4)	C10—C11	1.361 (4)
C1—C2	1.402 (4)	C10—H10	0.9500
C2—C3	1.387 (4)	C11—H11	0.9500
C2—H2	0.9500	C12—C13	1.349 (4)
C3—C4	1.376 (4)	C12—H12	0.9500
C3—H3	0.9500	C13—H13	0.9500
C4—C5	1.376 (4)		
			
C1—Sn1—C1^i^	180.0	C6—C5—C4	120.3 (3)
C1—Sn1—Br1	90.53 (7)	C6—C5—H5	119.8
C1—Sn1—Br1^i^	89.47 (7)	C4—C5—H5	119.8
C1—Sn1—Br2	89.64 (7)	C5—C6—C1	120.1 (3)
C1—Sn1—Br2^i^	90.36 (7)	C5—C6—H6	120.0
C1^i^—Sn1—Br1	89.47 (7)	C1—C6—H6	120.0
C1^i^—Sn1—Br1^i^	90.53 (7)	N1—C7—H7A	109.5
C1^i^—Sn1—Br2	90.36 (7)	N1—C7—H7B	109.5
C1^i^—Sn1—Br2^i^	89.64 (7)	H7A—C7—H7B	109.5
Br1—Sn1—Br1^i^	180.0	N1—C7—H7C	109.5
Br1—Sn1—Br2	88.981 (8)	H7A—C7—H7C	109.5
Br1—Sn1—Br2^i^	91.019 (8)	H7B—C7—H7C	109.5
Br1^i^—Sn1—Br2^i^	88.981 (8)	N1—C8—H8A	109.5
Br1^i^—Sn1—Br2	91.019 (8)	N1—C8—H8B	109.5
Br2—Sn1—Br2^i^	180.0	H8A—C8—H8B	109.5
C9—N1—C7	120.7 (2)	N1—C8—H8C	109.5
C9—N1—C8	121.0 (2)	H8A—C8—H8C	109.5
C7—N1—C8	118.2 (2)	H8B—C8—H8C	109.5
C12—N2—C11	121.0 (3)	N1—C9—C10	122.1 (2)
C12—N2—H2N	118 (3)	N1—C9—C13	121.0 (3)
C11—N2—H2N	120 (3)	C10—C9—C13	116.9 (2)
C6—C1—C2	118.9 (3)	C11—C10—C9	120.0 (3)
C6—C1—Sn1	120.3 (2)	C11—C10—H10	120.0
C2—C1—Sn1	120.71 (19)	C9—C10—H10	120.0
C3—C2—C1	120.0 (3)	N2—C11—C10	120.8 (3)
C3—C2—H2	120.0	N2—C11—H11	119.6
C1—C2—H2	120.0	C10—C11—H11	119.6
C4—C3—C2	120.2 (3)	N2—C12—C13	121.5 (3)
C4—C3—H3	119.9	N2—C12—H12	119.3
C2—C3—H3	119.9	C13—C12—H12	119.3
C3—C4—C5	120.4 (3)	C12—C13—C9	119.7 (3)
C3—C4—H4	119.8	C12—C13—H13	120.1
C5—C4—H4	119.8	C9—C13—H13	120.1
			
Br1^i^—Sn1—C1—C6	−130.5 (2)	C2—C1—C6—C5	1.7 (4)
Br1—Sn1—C1—C6	49.5 (2)	Sn1—C1—C6—C5	−175.7 (2)
Br2^i^—Sn1—C1—C6	−41.5 (2)	C7—N1—C9—C10	−1.6 (4)
Br2—Sn1—C1—C6	138.5 (2)	C8—N1—C9—C10	−178.0 (2)
Br1^i^—Sn1—C1—C2	52.2 (2)	C7—N1—C9—C13	178.9 (2)
Br1—Sn1—C1—C2	−127.8 (2)	C8—N1—C9—C13	2.6 (4)
Br2^i^—Sn1—C1—C2	141.2 (2)	N1—C9—C10—C11	−176.8 (3)
Br2—Sn1—C1—C2	−38.8 (2)	C13—C9—C10—C11	2.6 (4)
C6—C1—C2—C3	−1.5 (4)	C12—N2—C11—C10	−1.4 (5)
Sn1—C1—C2—C3	175.9 (2)	C9—C10—C11—N2	−0.9 (4)
C1—C2—C3—C4	−0.2 (4)	C11—N2—C12—C13	1.7 (5)
C2—C3—C4—C5	1.7 (4)	N2—C12—C13—C9	0.2 (5)
C3—C4—C5—C6	−1.5 (4)	N1—C9—C13—C12	177.1 (3)
C4—C5—C6—C1	−0.2 (4)	C10—C9—C13—C12	−2.3 (4)

Symmetry codes: (i) −*x*+1, −*y*+1, −*z*+1.

### Hydrogen-bond geometry (Å, °)

**Table:**

*D*—H···*A*	*D*—H	H···*A*	*D*···*A*	*D*—H···*A*
N2—H2N···Br1	0.88 (1)	2.79 (3)	3.385 (3)	126 (3)
N2—H2N···Br2	0.88 (1)	2.81 (3)	3.485 (3)	135 (3)
